# Joining Processes for Fibre-Reinforced Thermoplastics: Phenomena and Characterisation

**DOI:** 10.3390/ma15155454

**Published:** 2022-08-08

**Authors:** Juliane Troschitz, Benjamin Gröger, Veit Würfel, Robert Kupfer, Maik Gude

**Affiliations:** Institute of Lightweight Engineering and Polymer Technology, Technische Universität Dresden, Holbeinstraße 3, 01307 Dresden, Germany

**Keywords:** thermoplastic composite, multi-material, joining, forming phenomena, material structure, process analysis, computed tomography

## Abstract

Thermoplastic composites (TPCs) are predestined for use in lightweight structures, especially for high-volume applications. In many cases, joining is a key factor for the successful application of TPCs in multi-material systems. Many joining processes for this material group are based on warm forming the joining zone. This results in a change of the local material structure characterised by modified fibre paths, as well as varying fibre contents, which significantly influences the load-bearing behaviour. During the forming process, many different phenomena occur simultaneously at different scales. In this paper, the deformation modes and flow mechanisms of TPCs during forming described in the literature are first analysed. Based on this, three different joining processes are investigated: embedding of inserts, moulding of contour joints, and hotclinching. In order to identify the phenomena occurring in each process and to describe the characteristic resulting material structure in the joining zones, micrographs as well as computed tomography (CT) analyses are performed for both individual process stages and final joining zones.

## 1. Introduction

Continuous-fibre-reinforced plastics have a high potential for use in lightweight structures due to their good specific mechanical properties and the adjustable characteristics. In particular, thermoplastic composites (TPCs) enable both highly integrative part design and efficient large-scale production [1]. Moreover, TPCs are advantageous regarding recycling aspects at the end of life compared to thermoset systems. In order to exploit the load-bearing capability of TPC structures, appropriate joining technologies are required [2]. For this reason, a large number of different joining systems have been developed that are specially tailored to the properties of TPCs [3,4,5]. Thus, the warm formability of TPCs is advantageously exploited in many joining processes, such as the moulding of pins into TPCs [6,7,8,9], flow drill joining [10,11], thermoclinching [12,13,14], embedding of inserts [15,16,17,18], moulding of contour joints [19], and hotclinching [20]. All these processes are based on warm forming the joining zone. Therefore, they have in common a local change of the material structure. For many processes, modified fibre paths [7,15,18] as well as varying fibre volume contents (FVCs) and matrix-rich zones [11,21,22,23] are described as a result of the joining processes. The locally varying material structural properties in joining zones influence the load bearing behaviour and must, therefore, be taken into account when predicting the deformation and damage behaviour [15]. In this respect, relevant properties in the joining zone are, in particular, the locally varying fibre orientation and fibre content, defects (e.g., fibre damage and voids), and the mechanical properties of the materials resulting from the process (e.g., crystallization and degradation of the matrix).

**Figure 1:**
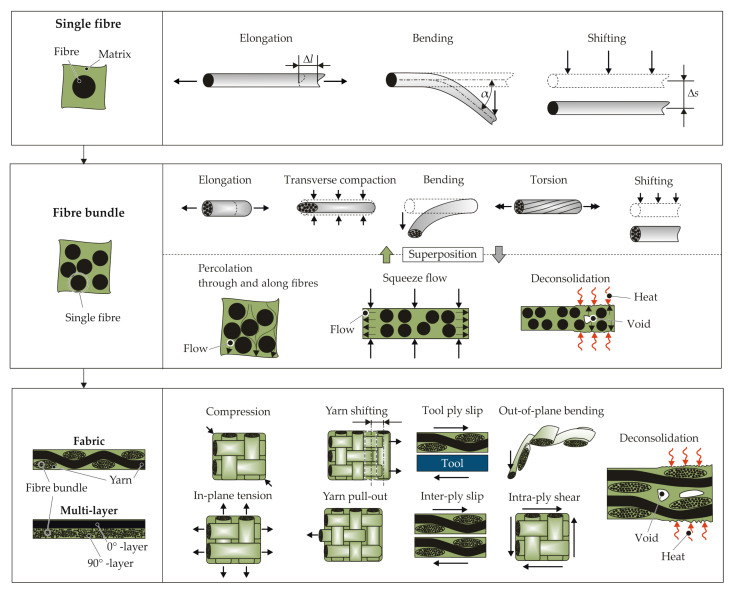
Scale-specific deformation modes and flow mechanisms of TPCs during forming.

The resultant material structure during the forming process depends on the material properties of the single constituents (e.g., matrix and fibre material, as well as textile architecture of the TPCs), the process parameters (e.g., temperature of the TPCs and the tools), and the design (e.g., geometry of the tools). Caused by the composition of two heterogeneous materials (fibre and matrix), a macroscopic point of view leads to a lack of information in terms of the occurring phenomena and material structure. Furthermore, the dimensions of the fibres (CF ⌀ 7 μm to 8 μm, GF ⌀ 14 μm to 20 μm) and yarns (cross-section approximately 2 mm × 4 mm) require a resolution on the mesoscopic or microscopic scale (Figure 1).

The forming process is determined by joining-induced forces, which lead to deformations of the yarns and interactions between the yarns (mesoscale); see Figure 1. These yarn deformations result in fibre interactions inside (microscale). Flow processes in the surrounding matrix lead to interactions between the fibres and matrix inside the yarns. These flow processes depend on the given process temperature. Two different temperature sections are identified:

I $T_{process}\geq T_{melt}$: Process temperatures above the melting temperature lead to independent matrix flow phenomena through the fibres (Figure 1, percolation, squeeze flow) and enable reorientations of the fibres in the surrounding matrix. As a result, the occurrence of fibre failures and cracks in the TPCs during forming can often be avoided.
II $T_{melt}>T_{process}$: Process temperatures below the melting temperature of the matrix inhibit the flow processes of the matrix (e.g., matrix percolation). When the temperature of the joining process is very low, the TPC behaviour is more brittle, caused by the supporting effect of the matrix. This can lead to fibre failures and cracks in the joining zone [24]. With rising temperature, the ductility of the matrix increases, while the supporting effect for the fibres decreases. Therefore, TPC sheets are often formed above the Vicat softening temperature to enable joining by plastic deformation. The fibres and the matrix can still not move independently. Squeeze flow occurs without percolation and can be described as a yielding.

Depending on the deformations and flow phenomena at a single fibre level, three different deformation modes can be described (Figure 1). Fibre elongation as well as shifting can be observed in the longitudinal direction of the fibre. Transversal to the fibre direction, bending and shifting can occur. The superposition of bending and shifting is often termed reorientation [18,25,26], rearrangement [7], or displacement [9]. In the following, the term reorientation is used.

On the fibre bundle scale, interactions (e.g., friction between fibres, slipping of fibres along the fibre direction) affect the material behaviour due to the large number of fibres in a yarn or a fibre bundle. A distinction of the fibre and fibre bundle behaviour is made by [27]. In forming processes above the melting temperature of the matrix, flow processes occur. In [28], the phenomena are investigated. Matrix percolation through and along the fibres and a transverse fibre flow, so-called squeeze flow, are observed on the microscale. These phenomena lead to an interaction between the molten matrix and fibres and also affect the material structure in terms of voids, matrix-rich zones, and fibre shifting, respectively bending. Moreover, when TPCs are heated above their melting temperature prior to forming, deconsolidation can occur. The driving mechanisms for deconsolidation are dissolved moisture expansion and released frozen-in fibre stresses [29,30].

The deformation and interactions of yarns in prepreg layers or dry fabrics at the mesoscopic scale are discussed in [31,32]. The deformation modes are in-plane tension, in-plane shear, ply–ply/ply–tool shear, and, transverse to the fibre direction, a compression [32] or compaction [31]. Both consider a ply bending in the out-of-plane direction. Furthermore yarn pull-out and yarn shifting are described as deformation modes in this context [31]. The deformation of the fabric, especially under tensile load, is characterised by structure deformation, also called yarn deformation, and afterwards, a fibre deformation [27] results in fibre strain (microscale).

All the phenomena described in the literature, which occur during the forming of TPCs, are summarized in Figure 1 and assigned to the different scales. The manufacturing of joining zones in TPCs is accompanied by these phenomena, leading to a change in the local material structure. However, so far, only individual phenomena or the final material structure have been described for the joining processes. Furthermore, at present, these complex superimposed deformation phenomena for those local forming processes cannot be numerically simulated. In order to describe such joining processes simulatively in the future, it is first necessary to analyse the single phenomena and their chronological occurrence in detail.

Therefore, joining technologies using thermally supported forming of TPCs are discussed in more detail in this work. The focus here is on processes where forming takes place above the Vicat softening temperature of the matrix. In order to investigate which phenomena occur during different processes, three technologies were selected, which differ in particular in terms of temperature management:

- For the embedding of inserts, the process temperature is above the melting temperature of the matrix (Temperature Section I) and an isothermal mould is used.
- Moulding of contour joints is also carried out above the melting temperature of the matrix (Temperature Section I), but in a variothermal mould.
- The hotclinching, on the other hand, is performed below the melting temperature of the matrix (Temperature Section II) using a variothermal mould.

## 2. Materials and Methods

### 2.1. Material Specification

Typical matrix systems for TPC applications with moderate thermal and mechanical requirements are polypropylene (PP) and polyamide 6 (PA6). Glass fibres (GFs) and carbon fibres (CFs) are usually used as reinforcement.

**Figure 2:**
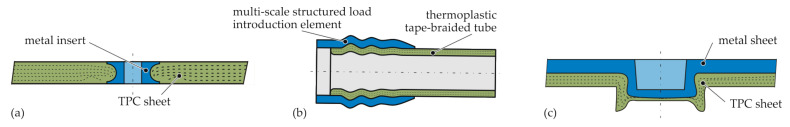
Schematic illustration of the investigated metal–TPC joints: embedded insert (**a**), contour joint (**b**), and hotclinched joint (**c**).

The joints with the different materials considered in this paper are shown in Figure 2. The TPC materials as well as the metal joining partners and auxiliary joining elements are summarised in Table 1.

The laminate layup for the embedding of inserts is made of unidirectional (UD) tapes, which are processed into TPC sheets in an autoclave process. The inserts are made of steel, and the height corresponds to the thickness of the laminate, so that they are flush with the laminate surface (Figure 2a). The laminate for moulding of contour joints is a hybrid layup of UD $0^{{}^{\circ}}$ layers and $\pm 30^{{}^{\circ}}$ layers with a 2 × 2 twill fibre architecture of braided slit tape with a width of 3 mm. For the hotclinching process, a stack of UD layers is used. The UD layers have stitch yarns in the 45${}^{{}^{\circ}}$-direction with a mass ratio of 3% for a better process handling of the individual yarns in the pre-consolidation steps.

### 2.2. Joining Processes

#### 2.2.1. Embedding of Inserts

**Figure 3:**
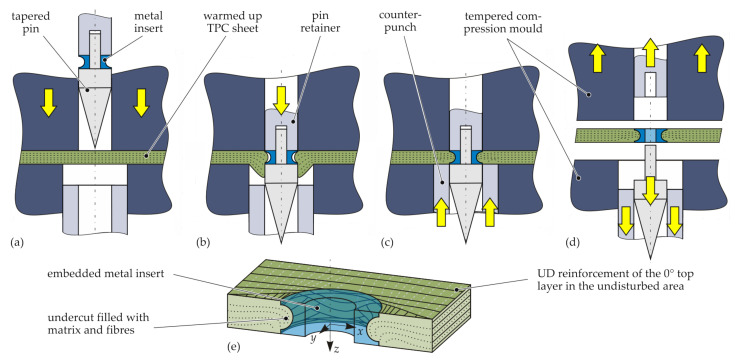
Schematic illustration of process-integrated embedding of inserts into the TPC during compression moulding (**a**–**d**) and the resulting joining zone (**e**).

Metal inserts can be embedded into TPCs during the part manufacturing process using the principle of moulding holes [17]. Thereby, the reinforcing fibres are not cut by punching or drilling, but reoriented by a tapered pin tool in a plasticised state of the TPC [15]. The process of embedding metal inserts into the TPC is schematically illustrated in Figure 3. First, the TPC sheet is heated above the melting temperature of the polymer matrix (for example, by an infrared heating device) and, then, quickly transferred into the open compression mould. Immediately after closing the tempered compression mould (Figure 3a), a tapered pin tool (consisting of a pin retainer and tapered pin) is shifted forward, forming a hole by displacing the reinforcing fibres and the still-molten matrix (Figure 3b). The two-part pin tool is equipped with a metal insert, which is precisely positioned flush with the composite’s surface. Subsequently, after the pin movement, the squeezed-out material is recompressed by a ring-shaped counterpunch (Figure 3c), whereby the undercut of the insert is filled with fibres and matrix material (Figure 3e). After cooling and solidification, the pin retainer can be retracted and the tapered pin is separated while the mould is opened. Finally, the shaped TPC part with the integrated metal insert can be demoulded (Figure 3d).

In this study, a pilot rig at the laboratory scale consisting of an infrared heating device (TPC temperature 210 °C) and a tempered steel mould ( 40 °C) was applied. The pin tool and the counterpunch were pneumatically actuated. The feed rate of the pin tool was 230 mm $s^{-1}$ and 160 mm $s^{-1}$ for the counterpunch. Thus, the embedding process (Steps b and c in Figure 3) takes less than 1 s.

#### 2.2.2. Moulding of Contour Joints

**Figure 4:**
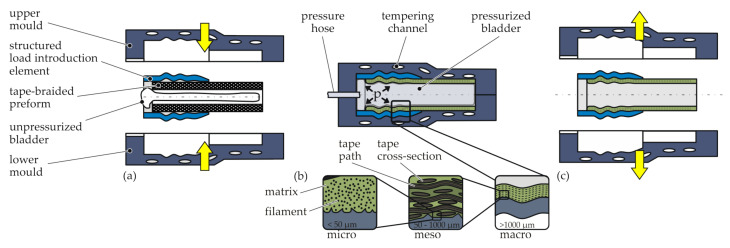
Schematic illustration of the integral-bladder-assisted moulding process: positioning (**a**), heating/consolidation and cooling/solidification (**b**), and release (**c**).

In the integral-bladder-assisted moulding process, two semi-finished parts are intrinsically joined during the manufacture of the composite structure. A tape-braided preform is inserted into a multi-scale-structured metallic load introduction (LI) element and positioned inside a mould (Figure 4a). By pressure application to the internal bladder and by melting the polymer matrix of the thermoplastic tape, the braided preform is moulded and consolidated in a moulding tool in the tubular section and into the contours of the LI element (Figure 4b). The temperature is controlled by a variothermal mould heating system. It heats and cools the mould, which conducts the heat to the preform. A form closure was created on different scales. The preform was moulded into the macro-contours of the LI element. Additionally, the LI element has a structured inner surface of $\pm 30^{{}^{\circ}}$ pyramids manufactured by knurling following DIN82 (RGE30) [33] with a division of 1 mm. Thereby, undulations are induced on the mesoscale, and by matrix percolation, small crevices are filled on the surface on the microscale. In this study, a CF-PA6 tape material was used, for which good moulding qualities can be achieved with a processing temperature of 230 ${}^{{}^{\circ}}$C and a consolidation pressure of 6 inside the internal bladder. After the matrix material is solidified in the cooling process, the hybrid structure is removed from the tool (Figure 4c).

#### 2.2.3. Hotclinching

**Figure 5:**
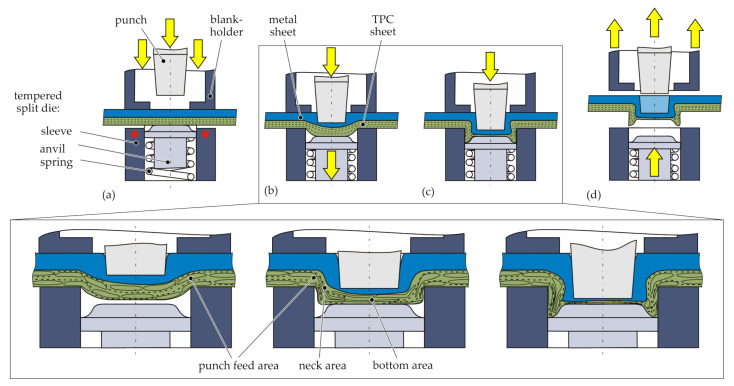
Schematic illustration of the hotclinching process: positioning, heating, and fixation (**a**), offsetting (**b**), upsetting and flow pressing (**c**), and releasing the finished joint (**d**).

The thermally supported clinching process (hotclinching) uses a tool concept consisting of a conventional punch, as well as a blankholder and a novel split die [20]. The die is assembled by a rigid tempered sleeve and a spring-loaded anvil (Figure 5). The tool concept enables a single-stage joining process for metal–TPC joints. It is an adaption of a conventional clinching process with a rigid die. The thermal support in the sleeve is generated by cartridge heaters. An advantage of the process is the omission of pilot holes or other preliminary steps before joining.

The schematic illustration of the process is given in Figure 5, including a detailed concept of the resultant material structure in the joining zone. The joining partners with the die-sided TPC are positioned between the tools (Figure 5a), and the TPC sheet is heated up to 180 ${}^{{}^{\circ}}$C (Temperature Section II). The heating process takes approximately 30 s. The process temperature is next to the Vicat softening temperature (for PA6, approximately 200 ${}^{{}^{\circ}}$C) and clearly below the melting temperature of around 220 ${}^{{}^{\circ}}$C. After reaching the process temperature, the blankholder moves downwards, followed by the punch with a velocity of 2 mm $s^{-1}$. The downward stroke of the punch leads to a deformation in the thickness direction. This offsetting phase ends when the anvil hits the mechanical stop 1 mm below the sleeve top (Figure 5b). Caused by the spring-loaded anvil, a counter-pressure between the sheets is applied. This leads to a stabilisation of the neck area of the punch-side sheet [18]. During the following upsetting phase, the bottom thickness of the TPC decreases with increasing punch movement [34]. The thinning effect while compression is characterised by a radial material flow. This results in the flow pressing phase (Figure 5c), where the undercut is generated. Finally, the finished joint can be released (Figure 5d). In total, the joining process without heating takes approximately 3 s.

### 2.3. Evaluation Methods

An analysis of the joining zone with imaging techniques is essential to identify and evaluate the process phenomena and their impact on the material’s structure. Micrograph analyses of cross-sections are often used for quality assessment or measurement of characteristic geometrical parameters [35]. In the present paper, the local material structure of the TPC in the joining zone was characterised by micrographs for both intermediate process stages and final joints.

Micrograph analyses have the disadvantage that the joining zone can only be evaluated in one section plane. Three-dimensional phenomena, such as fibre reorientations, can thus hardly be evaluated. For this reason, additional computed tomography (CT) analyses were carried out to investigate the joining zones. The specimens were investigated with a CT system V|TOME|X L450CT ( 300 kV micro-focus X-ray tube). The scanning parameters are summarized in Table 2. The reconstructed CT volumes were digitally processed in VG Studio Max (version 3.4, Volume Graphics GmbH, Heidelberg, Germany).

**Figure 6:**
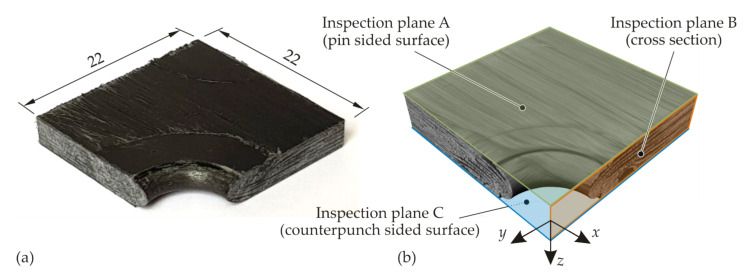
Analysis of a quarter joining zone with removed insert using CT: test specimen (**a**) and reconstructed 3D image (**b**). All dimensions are in mm.

To achieve a high image quality and to avoid artefacts, the joining zones with embedded inserts were sectioned. The insert was removed, and only the TPC of a quarter joining zone (22 mm × 22 mm × 4.3 mm) was scanned (Figure 6). To analyse the fibre orientation and the FVC, the fibre composite material analysis features of VG Studio Max were used. For this purpose, a threshold grey value was defined to distinguish between the fibre and matrix material. The threshold was adjusted in such a manner that the FVC outside the modified joining zone was equal to that of the undisturbed TPC (see Table 1). The scans of the hotclinching specimens were made 45 ${}^{{}^{\circ}}$ to the centre beam.

## 3. Process Phenomena

### 3.1. Embedding of Inserts

**Figure 7:**
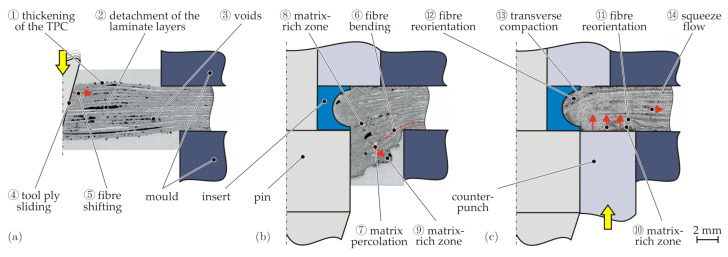
Analysis of the formation of the local material structure during the embedding process by microscopic analysis for different forming steps: pin movement (**a**), positioning of the insert in the laminate plane (**b**), and counterpunch movement (**c**).

In Figure 7, the intermediate process stages of the insert embedding process are presented. During the heating of the TPC in the infrared heating device, a deconsolidation of the structure takes place. This is characterised by an increase in the TPC sheet thickness (1), by local detachments of the individual laminate layers (2), as well as by the formation of voids within a layer (3). After the transfer of the TPC sheet into the mould and the closing process, this deconsolidation temporarily remains in the joining zone (Figure 7a).

The subsequent movement of the pin displaces the fibres and the matrix, causing friction and sliding between the pin tool and the TPC sheet (4). Thereby, the fibres are shifted (5), bent (6), and elongated, which results in a significant three-dimensional change of the fibre orientation (Figure 7b). Whereas shifting and bending can be observed directly in microscopic and CT analysis, elongation can only be detected indirectly. However, it can be stated that fibre elongation inevitably appears as the fibre path is enlarged during reorientation. In addition, it can be observed that percolation (7) occurs due to the flow of the matrix. As the fibres are tensioned, the matrix is squeezed out of the fibre bundles when the pin displaces the TPC. This leads both to areas inside the laminate that are only filled with matrix (8) and to matrix-rich zones on the counterpunch side (9). Several voids and local detachment of laminate layers from the previous process step partly remain (Figure 7b). When the pin reaches its end position, the insert is positioned flush in the laminate plane. Due to the fibre tension, the fibres previously in the forming zone reoriented and stretched are radially displaced into the undercut of the insert.

Afterwards, the material displaced in the thickness direction is pressed back into the laminate plane by the counterpunch (Figure 7c). Due to the pressure of the counterpunch, voids and local delaminations resulting from the deconsolidation at the beginning of the process are closed. Thereby, the gas in the voids is compressed and dissolved in the matrix [29]. The matrix-rich zones previously located on the counterpunch surface (9) are also pressed upwards (10). The fibres are partially shifted and bent when pressed back into the laminate plane, which is reflected in the cross-section by a reorientation of the fibres in the direction of the counterpunch feed (11). The pressure of the counterpunch ultimately also results in the complete filling of the insert’s undercut. In this area, a distinctive reorientation of the fibres (12) can be detected. During this process, a part of the matrix is pressed into the surrounding areas of the component (percolation), which have not yet been solidified either. Thus, in the area of the forming zone, there is no increase in the thickness of the TPC sheet. For this reason, it can be assumed that, locally, there is also transverse compaction (13) and squeeze flow (14) due to the counterpunch pressure.

**Figure 8:**
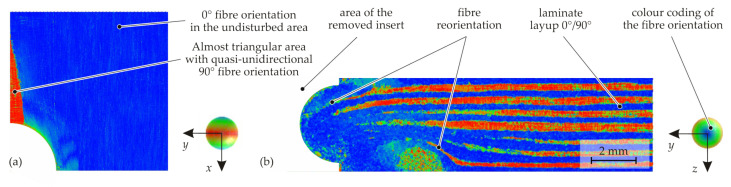
Analysis of the fibre orientation using CT data: pin-side surface-inspection Plane A (**a**) and cross-section-inspection Plane B (**b**).

For a three-dimensional analysis of the resulting material structure with locally varying fibre orientations and the FVC, CT analyses were carried out on the final joining zone. It can be identified that the fibres are not only reoriented in the thickness direction, but also in the laminate plane (Figure 8a), as in hole moulding processes [21,36]. This is accompanied by inter-ply slip. On the pin-side surface, the lateral in-plane displacement of the fibres results in an almost triangular area where the underlying 90 layer comes to the laminate surface. An examination of the cross-section shows that the initial layer structure of the composite disperses in the area near the insert (Figure 8b). The result is a complex material structure with three-dimensional fibre paths; the fibres are reoriented both in the laminate plane and in the thickness direction. Resulting fibre failures due to exceeding the elongation at break could neither be observed in the forming studies, nor in the final joining zone.

**Figure 9:**
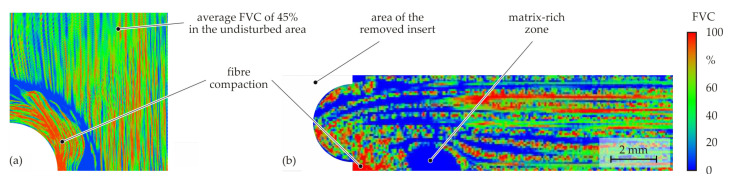
Analysis of the FVC using CT data: counterpunch-side surface-inspection Plane C (**a**) and cross-section-inspection Plane B (**b**).

As the micrograph analyses of the forming process showed, matrix-rich zones occur in the joining process. These areas were also detected in the CT analysis (Figure 9). Furthermore, it is evident that transverse compaction of the fibres also occurs. These areas are characterised by a locally very high FVC.

**Figure 10:**
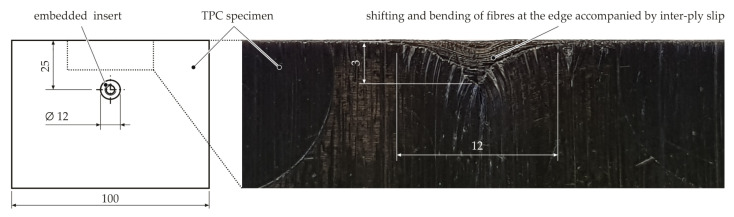
Analysis of the specimen edge for embedded inserts. All dimensions are in mm.

An examination of the specimen edge proves that inter-ply slip also occurs when the inserts are embedded. In Figure 10, it can be seen that the fibres are locally shifted in the longitudinal direction (see yarn pull-out in Figure 1) and bent, with single laminate layers sliding on one another. Thus, tool–ply slip also occurs locally at the outer layers. The appearance of the deformed edge area is strongly dependent on the fibre architecture and the edge distance of the insert.

### 3.2. Moulding of Contour Joints

**Figure 11:**
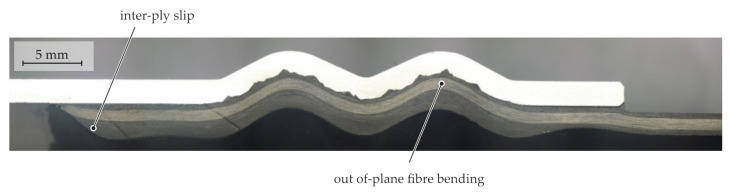
Photomicrograph of a longitudinal section of a multi-scale structured contour joint with an inner [$\left(\pm 70^{{}^{\circ}}\right)$_5_] bandage.

A multi-scale form closure is targeted with the presented hybrid contour joints. Photomicrographs provide insights into the moulding phenomena after the bladder-assisted moulding process. In Figure 11, a longitudinal section of a multi-scale contour joint with a knurled pyramid structure on the mesoscale is shown in a photomicrograph.

The main phenomena during the moulding of macroscopic structures is the forming of the TPC in the thickness direction, creating the desired form closure. By intra-ply shear deformation of the braided textile, expansion of the preform is enabled. During expansion in the bladder-assisted moulding process, fibre shifting on the fibre bundle scale occurs, as well as inter-ply slip and tool–ply slip on the multi-layer scale. These phenomena are necessary to mould the composite into the structures of the aluminium LI element.

**Figure 12:**
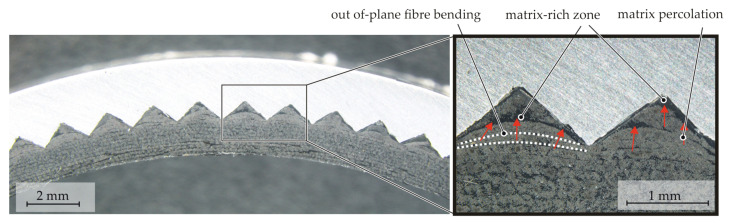
Photomicrograph of a cross-section of a meso-structured contour joint without a [$\left(\pm 70^{{}^{\circ}}\right)$_5_] bandage.

Depending on the shape of the meso-structure, certain geometries such as sharp edges or corners are not fully mouldable by the fibres via bending. In Figure 12, a photomicrograph of a section perpendicular to the axial direction of a meso-structured specimen without an inner [$\left(\pm 70^{{}^{\circ}}\right)$_5_] bandage is displayed. Bridging effects due to insufficient fibre bending occur in tight corners and grooves of the meso-structure pyramids. Due to matrix percolation, the cavities in the structured LI element are filled by the matrix material of the composite, which results in matrix-rich zones and a locally deviating FVC.

**Figure 13:**
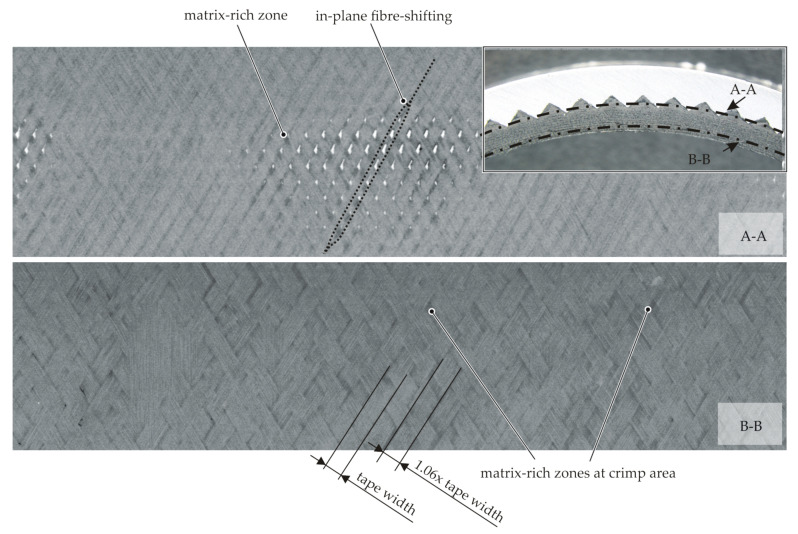
CT scan of the flattened surface of a meso-structured contour joint.

In Figure 13, two section views of a CT scan of the meso-structured contour joint are displayed. In Section A-A, the forming phenomena on the pyramid mesoscale are characterised by in-plane fibre shifting due to the pyramids piercing into the textile. By matrix percolation, the resulting gaps are filled. Gaps in the braided preform at the crimp area of the textile are filled by transverse squeeze flow and matrix percolation during the consolidation process when pressure is applied to the internal bladder, whereby the pockets in the crimp area are filled with fibres and matrix material (B-B). This results in an increase of the tape width.

### 3.3. Hotclinching

**Figure 14:**
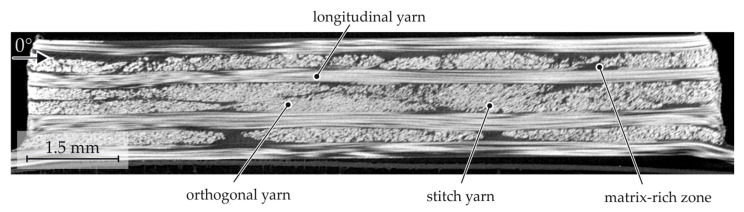
CT analysis of the initial material structure of the multilayered GF-PA6.

The initial material structure of the multi-layered GF-PA6 is characterised by unidirectional yarns with matrix-rich zones in between (Figure 14). The additional stitch yarns are used to fix the yarns during preconsolidation. The elliptical cross-sections of the yarns (orthogonal yarns) and the straight fibre path (longitudinal) can be clearly seen. Due to the stacked UD layers, fibre deformation modes dominate, whereas yarn deformation during joining cannot take place.

**Figure 15:**
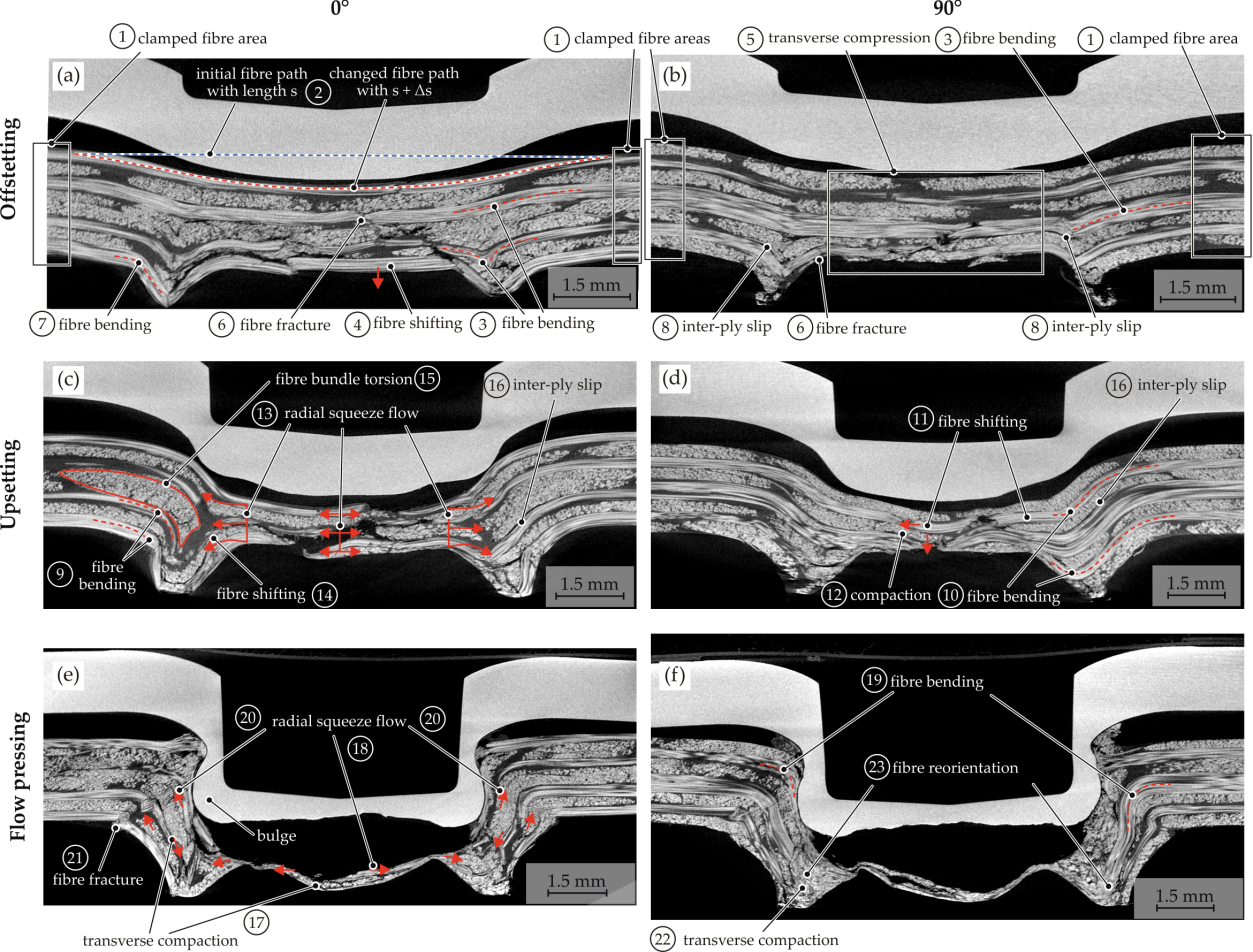
Resultant material structure and main phenomena for three phases of the hotclinching process: offsetting (**a**,**b**), upsetting (**c**,**d**), and flow pressing (**e**,**f**).

The hotclinching process operates in Temperature Section II below the melting temperature of the matrix. The temperature next to the Vicat softening temperature of the matrix just leads to a softening of the thermoplastic PA6 matrix and, therefore, to a more ductile material behaviour. This enables higher degrees of deformation without matrix failure and enables the joining by plastic deformations [34]. For detailed investigations of the phenomena occurring in the joining process, different stages of the joint formation were analysed with CT (Figure 15).

During heating, the fibres and the ductile matrix are clamped between the blankholder and the heated rigid die in the outer areas (1). The joining process starts when the punch moves downwards and offsets the sheets in the out-of-plane direction (Figure 15a,b). Due to the punch movement, the fibres are reoriented, the fibre path changes, and the length increases (2). This leads to fibre tension, resulting in elongation caused by the clamped fibres in the outer areas. The fibre sections in the punch feed area are bent in the out-of-plane direction (3), whereas the fibres in the bottom area are only shifted downwards (4). In the bottom area, the tension of the fibres is superimposed with the compression force in the thickness direction between the anvil and the punch-side sheet (5). The multi-layer sheet is compacted. In conclusion, regarding the fibre fractures in the bottom area (6), the complex stress state exceeds the fibre strength. In the ring channel area, the fibres are pressed in the anvil contour. Fibre bending and fibre fractures can be observed (7). Additionally, at the multi-layer scale, inter-ply slip can be seen in the ring channel area (8), caused by the different bending radii of each layer. This can lead to local delamination and layer separation.

**Figure 16:**
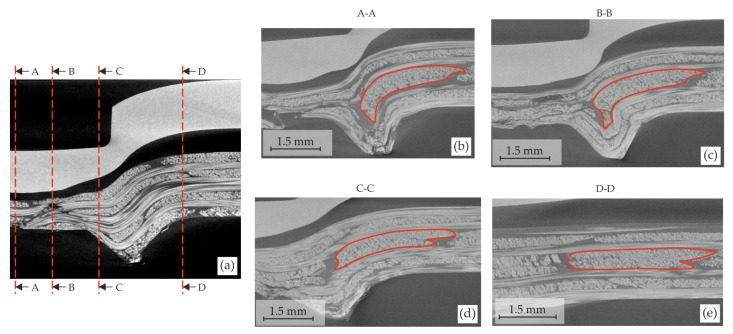
Change of the yarn cross-section along the fibre path characterised by the bending, shifting, and torsion of fibre bundles: definition of the cross-sections’ positions (**a**) and micrographs of the cross-sections (**b**–**e**). Torsion of the fibre bundle visualised by a red contour.

With the further downward stroke of the punch, a neck area is generated in the upsetting phase (Figure 15c,d). The fibre bending is still ongoing in the punch feed (9), neck, and ring channel area (10). Furthermore, the fibre shifting (11) and transverse compaction in the bottom area (12) increase. Due to the increasing joining force and the downward stroke, the free fibre endings (fibre fracture in offsetting phase) in the bottom area are displaced into the ring channel area by a radial squeeze flow (13). The radial flow shifts the orthogonal yarns in the neck area (14). Taking the further fibre path into account, this fibre shifting and bending lead to a fibre bundle torsion of the whole yarn (Figure 16). Especially in the orthogonal yarns, the phenomenon can be seen (Figure 15c). At the multi-layer scale, the inter-ply slip above the ring channel becomes larger with increasing punch stroke (16).

It can be stated that the fibre fracture in the bending areas also increases especially in the ring channel and the neck area (9 and 10), caused by the compression force and contour of the die and the anvil. The radii of joining tools are below the critical bending radius [37].

In the flow pressing phase (Figure 15e,f), the maximum transverse compaction in the bottom area (17) is achieved and the squeeze flow (18) in this area ends. Afterwards, the bulge with the undercut of the punch-sided metal sheet is formed (Figure 15e). The longitudinal fibres next to the bulge are bent (19). The forming increases the compression in the neck area, and a squeeze flow in the punch feed area and the ring channel begins (20). This squeeze flow in the neck area leads to fibre fractures by kinking (21). Furthermore, the fibres in the ring channel are shifted and bent due to the increasing transverse compaction (22). The inner material structure changes significantly in this area in comparison to the upsetting phase. The initial layers can no longer be identified, and the fibres are reoriented (23).

**Figure 17:**
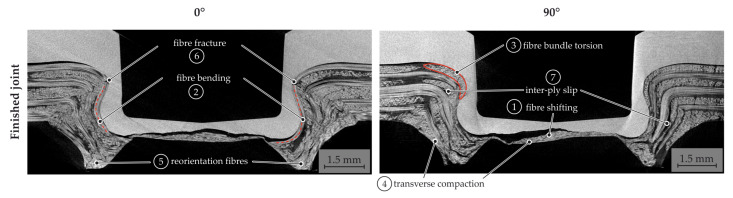
Resultant material structure of the finished hotclinched joint.

The CT analysis of the finished joint is shown in Figure 17. The resultant material structure after joining shows a three-dimensional reorientation of the fibres in the joining zone generated by the superposition of the individual phenomena. The main phenomena are fibre shifting (1) and bending (2) in the ring channel, as well as the neck area. Furthermore, the fibre bundle torsion is evident (3) in the final joint. Due to the compression and squeeze flow in the bottom area, a complex unspecific fibre reorientation (5) in the ring channel can be seen.

## 4. Discussion

All three joining processes considered result in a three-dimensional reorientation of the fibres in the joining zone across scales. The analyses of the joining zones were carried out on the fibre bundle and laminate scale; an evaluation on the single fibre level was not executed. However, it can be stated that a deformation at the fibre bundle scale also implies deformations of the single fibres.

Fibre bundle elongations cannot be determined with the applied characterisation methods (Table 3). However, due to friction between the fibres and between the fibres and the matrix, or the “clamping” of the reinforcement in areas with non-molten matrix, the fibres cannot move freely. Therefore, in general, in such local forming processes, elongation inevitably occurs when the fibre path is increased as a result of fibre reorientation [6,38].

In all three processes, transverse compaction occurs due to the out-of-plane forces on the joining zone. Depending on the matrix conditions (molten or not molten), this is accompanied by different flow processes (e.g., squeeze flow, matrix percolation). Deformation of fibre bundles can be detected successfully using CT analysis. Here, for example, the local fibre orientation in the joining zone can be evaluated qualitatively and quantitatively. As has been shown, all three processes lead to shifting and bending of fibre bundles. Torsion of fibre bundles was detected by CT analysis for hotclinching. In future works, fibre bundle torsion may be identified by analysing the paths of individual fibres in a fibre bundle during an in situ CT analysis of the forming process. When the breaking elongation of the fibres is exceeded in the process, fibre failure occurs. This could only be observed in the case of hotclinching. Here, the flexibility of the fibres is restricted due to the low temperature of the matrix during forming, which induces fibre failure in the joining zone.

In addition to the deformation of the fibre bundles, the flow processes of the matrix also occur during local forming if the matrix is flowable due to heating of the TPC (Table 4). For hotclinching, the process temperature is below the melting temperature of the matrix. Therefore, flow processes of the matrix according to [28] are not taking place. In contrast, flow processes of the matrix occur when embedding inserts or moulding contour joints. The flow processes of the matrix can only be demonstrated indirectly. It could be shown in micrographs, as well as CT examinations that there are matrix-rich zones and areas with increased FVC (see Figure 8 and Figure 12). These indicate that matrix percolation occurs. In addition, in all three processes, squeeze flow occurs due to the pressure on the joining zone.

Deconsolidation is observed only for embedding of inserts, because heating in an infrared heating device above the melting temperature of the matrix takes place without pressure on the TPC. Voids and detachments of laminate layers that occur during deconsolidation are closed in the pressure-impacted subsequent forming process, which was detected in the micrograph analyses of partially formed specimens (Figure 7). The heating of the TPC during the moulding of contour joints takes place under pressure, preventing deconsolidation. In the hotclinching process, no deconsolidation is detected due to the low temperature of the TPC.

The deformation and interactions of fibres and fibre bundles, as well as the matrix flow mechanisms at the microscale and mesoscopic scale lead to multiple deformation modes at the macroscopic scale, as also described in the literature (Figure 1). In all three considered joining processes, in-plane tension/compression, in-plane shear, ply–ply slip, and bending in the out-of-plane direction occur. In addition, fibre bundle pullout at the edge was detected for the embedding of inserts. Furthermore, there are interactions between the tools and the joining partners resulting in tool–ply slip in all three processes. This is influenced by friction and is particularly dependent on the temperature distribution and the forming speed.

The contributions of the individual phenomena during forming depend on the material properties, such as the textile architecture, the process parameters, such as the TPC temperature and tool velocity, and the design, such as the geometry of the tools or the structured surface.

## 5. Conclusions

In this paper, the process phenomena at different scales occurring during the forming of TPCs as described in the literature were analysed. On this basis, three different joining processes were investigated regarding the deformation modes and flow mechanisms of the TPC during forming. Although the joining processes were very different, especially with respect to the temperature of the TPC (below and above the melting temperature), it was shown that almost all the forming phenomena described in the literature can occur also during joining. The superposition of the forming phenomena on the different scales results in locally complex material structures with varying properties such as the fibre orientation and FVC. This local material structure affects the load-bearing behaviour of the joint. The numerical description of those joining processes with fibre–matrix interaction is not possible yet. For this purpose, it is necessary to reduce the complexity by focusing on the individual phenomena first. Within this work, a major step is made by describing complex forming processes with a superposition of single phenomena taking into account the common deformation theories of forming.

## Figures and Tables

**Table 1:** Specification of the utilised materials and semi-finished products (properties according to manufacturer’s specifications except ^(1)^ experimentally determined for the FVC of 44%).

		Embedding of Inserts	Moulding of Contour Joints	Hotclinching
TPC	Material	GF-PP	CF-PA6	GF-PA6
	Name	Celstran^®^ CFR-TP PP-GF70	Celstran^®^ CFR-TP PA6 CF60-03	Tepex^®^ dynalite 102- RGUDm317(8) 47%
	Configuration	UD [($0^{{}^{\circ}}$/$90^{{}^{\circ}}$)_8_]_s_	[$\pm 30^{{}^{\circ}}$_2_/$0^{{}^{\circ}}$_7_/$\pm 30^{{}^{\circ}}$_2_/$\pm 70^{{}^{\circ}}$_5_]	UD [($0^{{}^{\circ}}$/$90^{{}^{\circ}}$)_4_]_s_
	FVC	45%	48%	47%
	Thickness	4.3 mm	2 mm	2 mm
	Melting temperature	173 ${}^{{}^{\circ}}$C	220 ${}^{{}^{\circ}}$C	220 ${}^{{}^{\circ}}$C
	Tensile modulus (UD 0°)	34 GPa	100 GPa	33.6 GPa ^(1)^
	Tensile strength (UD 0°)	0.9 GPa	1.9 GPa	0.6 GPa ^(1)^
Metal	Material	steel 1.2210	EN AW-6060 T4	EN AW-6016 T4
	Thickness	4.3 mm	2 mm	1.5 mm
	Outer diameter	12 mm		
	Young’s modulus	210 GPa	68 GPa	70 GPa
	Tensile strength	0.7 GPa	0.15 GPa	0.12 GPa

**Table 2:** Specification of the CT scanning parameters.

	Embedding of Inserts	Moulding of Contour Joints	Hotclinching
X-ray voltage	80 kV	160 kV	100 kV
Tube current	250 μA	160 μA	220 μA
Exposure time	2000 ms	500 ms	500 ms
X-ray projections	2880 (8 per 1°)	1440 (4 per 1°)	1440 (4 per 1°)
Voxel size	13.6 μ m	41.7 μ m	8 μm

**Table 3:** Detected deformations on fibre bundle scale.

	Elongation	Transverse Compaction	Bending	Torsion	Shifting
Embedding of Inserts	n.a.	Occurring	Occurring	n.a.	Occurring
Moulding of Contour Joints	n.a.	Occurring	Occurring	n.a.	Occurring
Hotclinching	n.a.	Occurring	Occurring	Occurring	Occurring

**Table 4:** Occurring interactions of fibres and matrix during the joining processes.

	Percolation through and along Fibres	Squeeze Flow	Deconsolidation
Embedding of Inserts	Occurring	Occurring	Occurring
Moulding of Contour Joints	Occurring	Occurring	Not occurring
Hotclinching	Not occurring	Occurring	Not occurring

## Abbreviations

The following abbreviations are used in this manuscript:

CF	Carbon fibre
CT	Computed tomography
FVC	Fibre volume content
GF	Glass fibre
LI	load introduction
PP	Polypropylene
PA6	Polyamide 6
TPC	Thermoplastic composites
UD	Unidirectional
