# Correction: Troschitz et al. Joining Processes for Fibre-Reinforced Thermoplastics: Phenomena and Characterisation. *Materials* 2022, *15*, 5454

**DOI:** 10.3390/ma16124376

**Published:** 2023-06-14

**Authors:** Juliane Troschitz, Benjamin Gröger, Veit Würfel, Robert Kupfer, Maik Gude

**Affiliations:** Institute of Lightweight Engineering and Polymer Technology, Technische Universität Dresden, Holbeinstraße 3, 01307 Dresden, Germany

The authors would like to make the following corrections about the published paper [1]. The changes are as follows:

## 1. Correction in Table 1

In Table 1, there was a mistake in the laminate configuration of the embedded inserts. UD [(0°/90°)_8_]_s_ was written instead of the correct UD [(0°/90°)_4_]_s_. The corrected Table 1 appears below.

## 2. Correction in Figure Captions

There was a mistake in the captions for Figures 11 and 12. The use of externally provided images by Fraunhofer IWU was not mentioned. The correct figure captions appear below.

**Figure 11.** Photomicrograph of a longitudinal section of a multi-scale structured contour joint with an inner [(±70°)_5_] bandage. Photomicrograph provided by Fraunhofer IWU.
**Figure 12.** Photomicrograph of a cross-section of a meso-structured contour joint without a [(±70°)_5_] bandage. Photomicrograph provided by Fraunhofer IWU.

## 3. Addition of an Acknowledgement

Raik Grützner (Fraunhofer Institute for Machine Tools and Forming Technology IWU) was not included as an author in the original publication. The added acknowledgements section appears here.

**Acknowledgments:** The authors gratefully acknowledge the provision of photomicrograph images of the contour joint by Raik Grützner of the Fraunhofer Institute for Machine Tools and Forming Technology IWU.

The authors apologize for any inconvenience caused and state that the scientific conclusions are unaffected. The above corrections were approved by the Academic Editor. The original publication has also been updated.

## Figures and Tables

**Table 1:** Specification of the utilised materials and semi-finished products (properties according to manufacturer’s specifications except ^(1)^ experimentally determined for the FVC of 44%).

		Embedding of Inserts	Moulding of Contour	Joints Hotclinching
TPC	Material	GF-PP	CF-PA6	GF-PA6
	Name	Celstran® CFR-TP PP-GF70	Celstran® CFR-TP PA6 CF60-03	Tepex® dynalite 102-RGUDm317(8)/47%
	Configuration	UD [(0°/90°)_4_]_S_	[±30°_2_/0°_7_/±30°_2_/±70°_5_]	UD [(0°/90°)_4_]_S_
	FVC	45%	48%	47%
	Thickness	4.3 mm	2 mm	2 mm
	Melting temperature	173 °C	220 °C	220 °C
	Tensile modulus (UD 0°)	34 GPa	100 GPa	33.6 GPa ^(1)^
	Tensile strength (UD 0°)	0.9 GPa	1.9 GPa	0.6 GPa ^(1)^
Metal	Material	steel 1.2210	EN AW-6060 T4	EN AW-6016 T4
	Thickness	4.3 mm	2 mm	1.5 mm
	Outer diameter	12 mm		
	Young’s modulus	210 GPa	68 GPa	70 GPa
	Tensile strength	0.7 GPa	0.15 GPa	0.12 GPa
